# Five-Year Assessment of Time of Sputum Smears Conversion and Outcome and Risk Factors of Tuberculosis Patients in Central Iran

**DOI:** 10.1155/2015/609083

**Published:** 2015-01-14

**Authors:** Fatemah Behnaz, Mahmoud Mohammadzadeh, Golnaz Mohammadzade

**Affiliations:** ^1^Department of Infectious Diseases, Shahid Sadoughi University of Medical Sciences, Yazd, Iran; ^2^Shahid Sadoughi University of Medical Sciences, Yazd, Iran

## Abstract

*Objective*. To evaluate risk factors which influence sputum smear conversion, outcome, and trends of conversion of sputum smear during 5 years and compare outcomes in patients with different regimens. *Methods*. In a retrospective cohort study, all patients with sputum smear positive tuberculosis were evaluated for comorbidities and demographic, microbiological, and therapeutic data and outcome. Smear examinations were performed at the beginning, at 2 months for CAT I, at 3 months for CAT II, at the end of second month of maintenance phase, and at the end of treatment. *Results*. This study enrolled 211 sputum smear positive patients, but 189 patients who completed the intensive phase of treatment were evaluated. Sputum smear of 158 patients converted at the end of intensive phase (83.6). Univariate analysis indicated that the risk of a persistent positive smear at the end of intensive phase was greater in diabetic patients ((odds 4.038, 95% CI 1.123–14.516) *P* = 0.033), and also a 3+bacillary load had risk of 2.933-fold ((95% CI 1.278–6.732) *P* = 0.011). Overall rate of unfavorable outcome was 20.9%. Factors associated with unfavorable outcome were age (*P* value 0.000), male gender (*P* value 0.027), diabetes (*P* value 0.000), and delayed conversion of sputum at the end of intensive phase (*P* value 0.000). Outcome for different regimens was not different significantly. Two specimens were isoniazid resistant. *Conclusions*. We suggest supervised treatment and care for diabetic patients and those with higher bacillary load. Paying attention to early diagnosis of tuberculosis in the elderly to reduce poor outcome and further measures to prevent transfer-out could improve the success rate.

## 1. Introduction

Tuberculosis (TB) remains a global health problem. In 2011, there were an estimated 8.7 million new cases of TB (13% coinfected with HIV) and 1.4 million people died from TB. Between 1995 and 2011, 51 million people were successfully treated for TB in countries that had adopted the WHO strategy, saving 20 million lives [1]. Successful control of tuberculosis (TB) depends on early and effective control of the transmission of* Mycobacterium tuberculosis* from infectious patients. Sputum smear conversion at the end of the intensive phase of treatment is used as an important early predictor of treatment success [2–4]. WHO continues to recommend performing smear microscopy at this stage because a positive smear should trigger an assessment of the patient, as well as additional sputum monitoring [1]. The sputum conversion results are used both for management of patients and for monitoring programmed performance [5]. Several studies evaluated risk factors such as age, smear grading, gender, influence of directly observed therapy (DOT), and associated comorbid conditions like HIV infection and diabetes mellitus among tuberculosis patients [5–8]. However, interest in geographic differences in response to TB treatment dates back to 1956 when Fox et al. compared clinical, radiographic, and microbiological outcomes in patients from Britain and Uganda [9]. In recent years Tuberculosis Trials Consortium Study 28 found later sputum culture conversion and lower rates of conversion in liquid media in African patients compared to non-African patients [10]. So, we studied risk factors which influence smear conversion and evaluated trends of conversion of sputum smear during a period of 5 years and compared outcomes in patients with pulmonary TB who received different regimens.

## 2. Materials and Methods

### 2.1. Study Design and Population

In a retrospective cohort study, all patients with sputum smear positive pulmonary TB who were referred to the Yazd Health Center from March 2007 to March 2012 were considered eligible for inclusion in this study. Comorbidities and demographic, microbiological, and therapeutic data were collected from patients' medical record. Based on the National Plan against Tuberculosis, all new smear positive pulmonary tuberculosis patients were classified as category (CAT I) regimen and all patients who were retreated were classified as CAT II regimen. Ethical clearance was obtained from the University Ethics Committee. This study did not need ethical consent.

### 2.2. Mycobacterial Examinations: Sputum Smear and Culture

Based on the National Plan against Tuberculosis, smear examinations were performed at the beginning, at 2 months for CAT I, 3 months for CAT II, at the end of second month of maintenance phase, and at the end of treatment. Culture and antimicrobial susceptibility were performed only for patients who were smear positive after three months of initiation of treatment. Sputum smears were examined for AFB by microscopy and Ziehl-Neelsen staining and graded by standard criteria and equivalent: 1–9 AFB/100 fields (1+); 1–9 AFB/10 fields (2+); and 1–9 AFB/field (3+) [11].

Smear conversion was defined as 2 consecutive negative samples. Smear and culture conversion time was defined as duration from the beginning of treatment to the date of the first negative sample. Delayed smear conversion was defined as persistent smear positivity after 2 months of treatment for patients in CAT I and 3 months for CAT II.

### 2.3. Treatment and Monitoring

A 6-month standard treatment based on international consensus 1 consists of intensive phase isoniazid (H), rifampicin (R), pyrazinamide (Z), and ethambutol (E) daily in the first 2 months; if the smear were positive, the intensive phase is extended by one more month for that patient. Treatment is followed by continuous phase (4 months), daily H and R, and can be prolonged in patients with concomitant hepatic and/or renal diseases and in case adverse effects occur. All new pulmonary TB patients were treated with this regimen irrespective of associated comorbid conditions. DOTS strategy was done in the intensive phase of all patients. All recurrent pulmonary TB patients were treated with HRZES daily for 2 months, HRZE daily for 1 month (intensive phase), and HRE daily for 5 months (continuous phase). Patients who were treated as CAT II were completely supervised for the entire course of treatment by DOTS strategy.

Outcome was defined as follows: cure: a patient whose sputum smear or culture was positive at the beginning of the treatment but who was smear or culture negative in the last month of treatment and on at least one previous occasion.

#### 2.3.1. Definitions

*Treatment Failure*. Treatment failure is defined as a patient whose sputum smear or culture is positive at 5 months or later during treatment. Also included in this definition are patients found to harbor a multidrug resistant (MDR) strain at any point of time during the treatment, whether they are smear negative or positive.

*Died*. “Died” was defined as a patient who dies from any cause during the course of treatment.

*Transfer-Out*. Transfer-out is a patient who has been transferred to another recording and reporting unit and whose treatment outcome is unknown [12].

### 2.4. Statistical Analysis

Statistical analysis was performed using the SPSS version 17.0 software. All probabilities were two-tailed and *P* values <0.05 were regarded as significant.

Data were described as mean with standard deviation. The Chi-square test or the Fisher exact test was used to compare categorical variables whenever appropriate. Delayed smear conversion was a dichotomous dependent variable. Variables that were statistically significant and biologically plausible in univariate analysis were entered into a logistic regression model with forward stepwise conditional in order to identify the factors independently associated with that outcome. The odds ratios (OR) and 95% confidence intervals (CI) were determined.

## 3. Results

All of the two hundred and eleven sputum smear positive patients who have been treated during the time of study were retrospectively enrolled. The patients' mean age was 52.93 ± 22.19 (range: 2–95 years), and 114 patients were male, 54%. Study population included 110 Iranian and 101 non-Iranian patients, most of whom were immigrants from Afghanistan. Frequency of diabetes mellitus among patients with pulmonary TB in this study was 9.5%, which was the most common underlying comorbidity.  Table 1 shows demographic characteristics, grading of sputum smear positivity at the initiation of treatment, anti-TB treatment regimens, treatment category, and comorbidities of enrolled patients at baseline.

Of 211 patients who were enrolled into the study, 22 had no record of sputum conversion at the end of the intensive phase; reasons were as follows: 9 patients were transferred out and 13 patients deceased during intensive phase of treatment. Of 189 patients who completed the intensive phase of treatment, sputum smear of one hundred and fifty-eight patients converted at the end of intensive phase (83.6%). Conversion rates of patients in CAT I were 144/173 (83.23%) and of patients in CAT II were 14/16 (87.5%) and conversion rate was not associated with category of treatment (*P* value = 0.660). The higher smear grading was associated with delayed conversion time (1+: 11.2%, 2+: 12.5%, and 3+: 30% (*P* = 0.01)). Diabetes mellitus and other comorbidities were associated with delayed conversion time (*P* = 0.012), whereas there was no association between age, sex, nationality, treatment regimens, and category of treatment with delayed conversion time in the present study (Table 2). In nine patients who had positive sputum culture and antimicrobial susceptibility, only two specimens of sputum were isoniazid resistant.

The outcome at the end of treatment for 211 enrolled patients was as follows: cure was achieved for one hundred and sixty-seven patients (79.1%) and unfavorable outcome during this study was 20.9% including treatment failure for 9 patients (4.3%), eight of whom were new smear positive cases, and death for nineteen patients (9%) during period of their treatment some of them due to diseases such as myocardial infarction, cerebrovascular accident, respiratory failure due to asthma, and COPD. The mean age of patients who died was 64 ± 18.86. From 16 patients (7.6%) who were transferred out, 9 patients returned to Afghanistan and health services could not trace them to find their outcomes. Factors associated with unfavorable outcome included age (*P* value 0.000), sex (*P* value 0.027), accompanying disease (*P* value 0.000), and sputum conversion at the end of intensive phase ((*P* value 0.000), Table 3). As is shown in Table 3 there was not any association between nationality, treatment regimen, initial sputum grading, or category of treatment and outcome in the present study.

As is shown in Table 4, after omitting patients who deceased and were transferred out, factors associated with failure included initial sputum grade (*P* value 0.004), diabetes mellitus (*P* value 0.00), and sputum conversion at the end of intensive phase (*P* value 0.00).

Univariate analysis indicated that the risk of a persistent positive smear at the end of 2nd month was greater in diabetic patients ((odds 4.038, 95% CI 1.123–14.516) *P* = 0.033), and also a 3+bacillary load had risk of 2.93-fold ((95% CI 1.28–6.73) *P* = 0.011) (Table 5).

**Figure 1:**
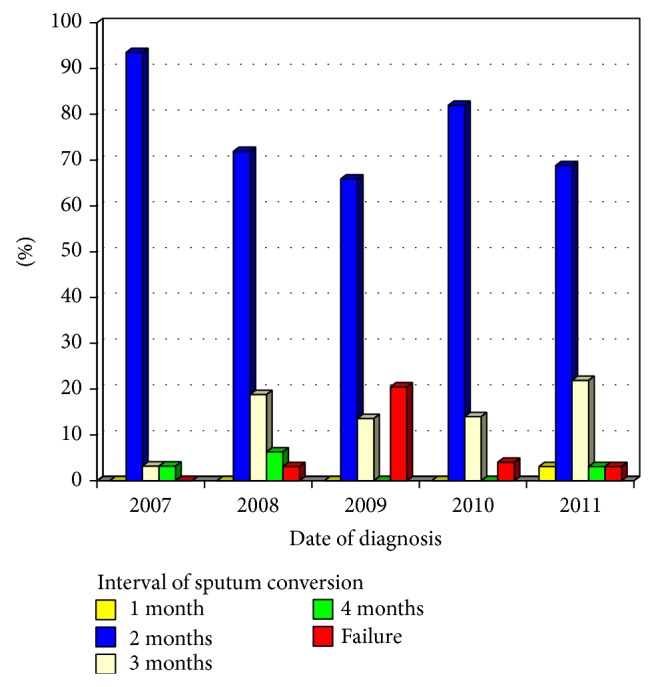
Time of sputum smear conversion of pulmonary tuberculosis regarding years of study.

**Figure 2:**
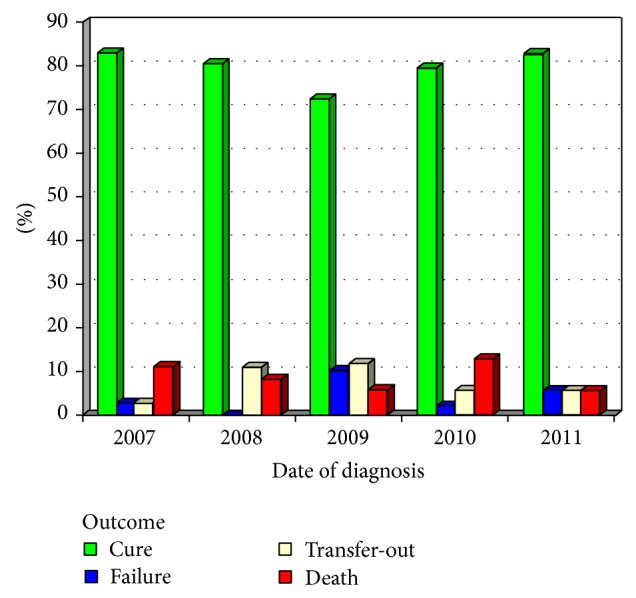
Outcome of pulmonary tuberculosis patients regarding years of study.

Figure 1 illustrates sputum conversion rates and Figure 2 shows outcome during years of study.

## 4. Discussion

In pulmonary tuberculosis, the assessment of response to therapy is evaluated by disappearance of acid-fast bacilli (AFB) from sputum smear and the conversion of culture to negative [13]. Sputum smear of one hundred fifty-eight patients (83.6%) converted at the end of intensive phase in the present study. The conversion rate reported from India at the end of 2nd month was 84% [14].

Another study from Kuwait reported conversion rate of 88.5% in smokers and 94.2% in nonsmokers [15]. A study from Taiwan revealed that 11.1% of patients remained smear and culture positive after 2 months of treatment [16]. So the conversion rate in the present study is in the range of the mentioned studies. It has been known that the proportion of smear positive patients at the end of the intensive phase is a predictor of treatment success. So conversion rate of our patients is acceptable and indicates high compliance of patients, as well as the dosage and quality of TB medications.

The conversion rate was not different between Iranians and non-Iranians, most of whom were immigrants from Afghanistan and lived in Iran for a long time. We found higher initial smear grading which was associated with delayed conversion time (*P* = 0.01). This finding was observed in previous studies [7, 14, 15, 17].

Identification of risk factors for persistent smear positivity after 2 months of treatment which can predict treatment failure is very important [18].

Frequency of diabetes mellitus (DM) among patients with pulmonary TB in this study was 9.5% and was the most common underlying comorbidity. The data presented in this study showed that patients with DM and TB have more delayed sputum conversion and a higher probability of treatment failure. The risk of nonconversion of sputum at the end of intensive phase of treatment associated with DM in our study was 2.44-fold (95% CI 1.11–5.40). Treatment outcomes in patients with TB and DM have been a subject of debate. In a study performed in India smear conversion was similar in diabetic and nondiabetic patients [17], in contrast to another study conducted in México which identified diabetes as an independent risk of failure to treatment [19]. Defects in the immune system of patients with active TB and DM have been reported, including reductions in the activation of alveolar macrophages and the capacity to produce interleukin [20, 21], in addition to some degree of impaired limited numbers of diabetic patients' gastrointestinal drug absorption even in the absence of clinical gastroparesis [22]. This can explain delay of sputum smear conversion and higher unfavorable outcome in the present study. However, due to limited number of diabetic patients in this study, it could not be generalized.

Treatment outcome results serve as a tool to control the quality of TB treatment provided by the health care system. In our study, the outcome at the end of treatment was cure (79.1%). This is close to the WHO target of success rate of 85% of all smear positive cases. However, subgroups of patients contributing to unfavorable outcome should specifically be considered. In the present study 15 patients (7.1%) were transferred out; majority of them left this country to their own country (Afghanistan) while they were on treatment, so we have no information about their outcomes. This is a common problem in treatment of Afghani patients and efforts should be made to ensure the continuity of treatment for patients who move out of Iran for prevention of drug resistance. Six patients out of 15 transferred-out patients belonged to younger age group which explains poor outcome in this group. The death rate in our study was 9%; most patients who died were older than cured patients, and some of them also had other illnesses. It is difficult to know to what extent the death of patients whose only cause of death was TB could have been prevented. But because most of them happened during early phase of treatment, it might be due to delay in their diagnosis and treatment.

Nine patients had treatment failure (4.3%); most of them were new smear positive. Drug susceptibility at the beginning of treatment is not routine in our country, so this can be due to primary or secondary resistance. Limited culture and drug susceptibility have been done during this study, not indicating multiple drug resistance. However, failure also indicates problem in quality of TB treatment and must be considered in the program of TB control.

In our study men had more unfavorable outcome, because they had more transferred out and failure. Most of them returned to their initial country and we do not know their outcome and probably the rest of them did not have good adherence to treatment, so health care workers must give more information at the initiation of treatment about duration of therapy and importance of adherence to that. Efforts should be made to ensure the continuity of treatment for patients who move out of the country and, if possible, to allow them to complete their course of treatment, even if they have to leave the country later.

Time to sputum smear conversion was similar during the study and differences in outcome cannot be attributed to improved/worsening of TB treatment practices in our setting, because TB control program in our country did not change during the period in which the study subjects have been treated.

## 5. Conclusions

Our analysis showed that 16.4% of tuberculosis patients remained smear positive at the end of intensive phase. Risk factors for persistent positive smear at 2 months were diabetes and higher bacillary load at the beginning of treatment. The outcome at the end of treatment was cure of 79.1% of patients. Factors associated with unfavorable outcome included younger age, male gender, diabetes, and delayed conversion of sputum at the end of intensive phase. We conclude that intensified treatment and precautions for TB patients with mentioned risk factors, early diagnosis of TB in elderly patients to reduce the death rate, and further measures to prevent transfer-out could improve the success rate further.

## Figures and Tables

**Table 1:** Comorbidities and demographic and bacteriological variables of sputum smear positive pulmonary tuberculosis patients in Yazd (*n* = 211).

Characteristic		Number %
Sex	Male	114 (54)

Age	≤50	86 (40.7)
	>50	125 (59.3)

Nationality	Iranian	110 (52.1)
	Non-Iranian	101 (47.9)

Initial sputum grading	1+	122 (57.8)
	2+	36 (17.1)
	3+	53 (25.1)

Accompanying disease	DM	20 (9.5)
	HIV	4 (1.9)
	Malignancy	1 (0.5)
	Transplant	2 (0.9)
	Others^*^	10 (4.7)

Treatment regimen	HRZE	185 (87.7)
	HRZES	18 (8.5)
	HRE	6 (2.8)
	HZE	2 (0.9)

Treatment category	CAT I	191 (90.5)
	CAT II	20 (9.5)

^*^Corticosteroid therapy (3), COPD (3), chronic renal failure (2), inherited cell mediated immunodeficiency (1), and cirrhosis (1). H: isoniazid; R: rifampicin; Z: pyrazinamide; E: ethambutol; S: streptomycin.

**Table 2:** Impact of comorbidities and demographic, microbiological, and therapeutic variables on sputum smear conversion at the end of the intensive phase (*N* = 189).

Characteristic		Sputum conversion status		*P* value
		Conversion number (%)	Nonconversion number (%)	
Sex	Male	83 (83.8)	16 (16.2)	0.925
	Female	75 (83.3)	15 (16.7)	

Age	≤50	60 (80)	15 (20)	0.279
	>50	98 (86)	16 (14)	

Nationality	Iranian	84 (85)	15 (15)	0.626
	Non-Iranian	74 (82.2)	16 (17.8)	

Initial sputum grading	≤2+	122 (88.4)	16 (11.6)	0.003
	3+	35 (70)	15 (30)	

Accompanying disease	DM	14 (70)	6 (30)	0.033
	Otherwise healthy (No comorbidity)	136 (87.8)	19 (12.2)	

Treatment regimen	HRZE	139 (83.7)	27 (16.3)	0.625
	HRZES^*^	13 (86.7)	2 (13.3)	
	HRE	4 (66.7)	2 (33.3)	
	HZE	2 (100)	0 (0)	

Treatment category	CAT I	144 (83.2)	29 (16.8)	0.660
	CAT II	14 (87.5)	2 (12.5)	

HRZES^*^: isoniazide, rifampicin, pyrazinamide, ethambutol, and streptomycin.

**Table 3:** Impact of comorbidities and demographic, microbiological, and therapeutic variables on outcome of patients (*N* = 211).

	Outcome					
Characteristic		Favorable	Unfavorable			*P* value
		Cure	Failure	Death	Transfer-out	
Sex	Male	83 (72.8)	6 (5.3)	11 (9.6)	14 (12.3)	0.027
	Female	84 (86.6)	3 (3.1)	8 (8.2)	2 (2.1)	

Age	≤50	65 (75.7)	2 (2.3)	4 (4.6)	15 (17.4)	0.000
	>50	102 (81.6)	7 (5.6)	15 (12)	1 (0.8)	

Nationality	Iranian	88 (80)	6 (5.5)	10 (9.1)	6 (5.5)	0.858
	Non-Iranian	79 (79)	3 (3)	9 (9)	9 (9)	

Initial sputum grading	≤2+	128 (81)	3 (1.9)	15 (9.5)	12 (7.6)	0.398
	3+	39 (75)	6 (11.5)	4 (7.7)	3 (5.8)	

Accompanying disease	DM	15 (75)	5 (25)	0 (0)	0 (0)	0.000
	Other comorbidities	10 (58.8)	0 (0)	6 (35.3)	1 (5.9)	
	No comorbidity	142 (81.6)	4 (2.3)	13 (7.5)	15 (8.6)	

Treatment regimen	HRZE	147 (80)	8 (4.3)	17 (9.2)	12 (6.5)	0.881
	HRZES^*^	13 (72.2)	1 (5.6)	1 (5.6)	3 (16.7)	
	HRE	59 (83.3)	0 (0)	1 (1.67)	0 (0)	
	HZE	2 (100)	0 (0)	0 (0)	0 (0)	

Treatment category	CAT I	153 (80.5)	8 (4.2)	17 (8.9)	12 (6.3)	0.53
	CAT II	14 (70)	1 (5)	2 (10)	3 (15)	

Sputum conversion	2 m for CAT I, 3 m for CAT II	146 (92.4)	2 (1.26)	4 (2.5)	6 (3.8)	0.000
	>2 m and >3	21 (67.8)	7 (28.6)	2 (6.4)	1 (3.2)	

^*^Isoniazide, rifampicin, pyrazinamide, ethambutol, and streptomycin.

**Table 4:** Impact of comorbidity and demographic, microbiological, and therapeutic variables on failure.

Characteristic		Outcome		*P* value
		Cure	Failure	
Sex	Female	84	3	0.32
	Male	83	6	

Age	≤50	65	2	0.31
	>50	102	7	

Nationality	Iranian	88	6	0.41
	Non-Iranian	79	3	

Initial sputum grading	≤2+	127	3	0.004
	3+	40	6	

Accompanying illness	Diabetic	15	5	0.00
	Otherwise healthy	152	4	

Treatment category	CAT I	153	8	0.775
	CAT II	14	1	

Sputum conversion	2 m for CAT I, 3 m for CAT II	146	2	0.00
	>2 m and >3	21	7	

**Table 5:** Risk estimate of nonconversion of sputum smear at the end of intensive phase.

Variable	Odds	95% CI	*P*
Age			
>50/≤50	0.694	0.291–1.658	0.412
Sex			
Female/male	1.048	0.462–2.377	0.910
Nationality			
Non-Iranian/Iranian	1.325	0.566–3.099	0.412
Initial sputum grading 3+/≤2+	2.933	1.278–6.732	0.011
Diabetic/other comorbidities			
No comorbidity	4.038	1.123–14.516	0.033
